# Predictors of Prosocial Behavior among Chinese High School Students in Hong Kong

**DOI:** 10.1100/2012/489156

**Published:** 2012-07-31

**Authors:** Andrew M. H. Siu, Daniel T. L. Shek, Frank H. Y. Lai

**Affiliations:** ^1^Department of Rehabilitation Sciences, The Hong Kong Polytechnic University, Hong Kong; ^2^Department of Applied Social Sciences, The Hong Kong Polytechnic University, Hong Kong

## Abstract

This study examined the correlates and predictors of prosocial behavior among Chinese adolescents in Hong Kong. A sample of 518 high school students responded to a questionnaire containing measures of antisocial and prosocial behavior, prosocial norms, pragmatic values, moral reasoning, and empathy. Preliminary analyses showed that there were gender differences in some of the measures. While correlation analyses showed that parental education, prosocial norms, pragmatic values, moral reasoning, and empathy were related to prosocial behavior, regression analyses showed that prosocial norms, pragmatic values, and empathy dimensions (personal distress and empathy) were key predictors of it. The findings are largely consistent with theoretical predictions and previous research findings, other than the negative relationship between personal distress and prosocial behavior. The study also underscores the importance of values and norms in predicting prosocial behavior, which has been largely neglected in previous studies.

## 1. Introduction

Prosocial behaviors are actions that aim to fulfill another person's need for support or to promote and sustain a positive benefit for them [1, 2]. In everyday life, this involves actions such as donating, sharing, comforting, expressing sympathy, helping, and providing physical assistance and support to others, often at a cost to oneself [3, 4]. Interestingly, individual prosocial behavior contributes not just to others' well-being, but also one's own. Various studies show that through helping and volunteering, young people can satisfy their own needs, learn about and express their values, understand the world gain career-related experience, and strengthen social competence and relationships [4–7]. Accordingly, interest in the study of prosocial behavior is growing around the world.

The cultivation of prosocial behavior has long been an important objective of compulsory education and youth development programs. Prosocial development is closely linked to various positive developmental outcomes for young people including academic success, positive self-worth, positive relationships with others, and higher social competence [4–6]. On the community or societal level, prosocial behaviors such as cooperation, taking responsibility and team work are crucial to the effective functioning of work and social interactions. Prosocial and altruistic behavior like volunteering or care giving could be a major source of a society's human capital.

In contrast to the abundance of western research on adolescent prosocial behavior, very few studies have been done with Chinese adolescents. In addition, while there are many studies on antisocial and deviant behaviors among adolescents, comparatively fewer look at prosocial behavior, including the predictors of it in adolescents. For example, Ma and colleagues [8, 9] show that several factors influence prosocial behavior in adolescents, including peer and teacher influences; family, school, and social environment; individual achievement. Although these studies are pioneering, they do not explore intrapersonal competencies such as prosocial norms, pragmatic values, moral reasoning, and empathy.

It is likely that prosocial development has largely taken shape by mid-to-late adolescence. By then, young people are likely to have developed a set of values to guide their behavior. Unfortunately, few studies have examined how personal values may influence prosocial behavior among young people. In this study, it is hypothesized that the adoption of prosocial norms and rejection of pragmatic values will play a part in determining prosocial behavior of young people. Prosocial norms are standards and beliefs, or the set of shared social expectations of healthy, ethical, appropriate, and culturally desirable actions, that promote prosocial behavior and minimize health risks [10, 11]. The label pragmatic values are used in this study to refer to values that are self-centered, materialistic, and instrumental in achieving one's own ends rather than others', such as one's own achievement, satisfaction, happiness, security or power [12, 13]. Furthermore, as moral reasoning is intimately related to prosocial behavior, it can also be hypothesized that a higher level of the former will be related to a higher level of the latter.

There are also theoretical accounts proposing that individual differences in prosocial development are closely linked to empathy-related constructs like sympathy, personal distress, perspective and role taking, social awareness, and moral reasoning [3, 14, 15]. Many of these competencies emerge in early life and continue to develop over childhood and adolescence. By the time young people reach late adolescence, they have passed the peak of their physical growth and become sexually mature and are starting to consolidate their identity. The rapid development of executive functioning, such as deductive reasoning and information processing, enables them to become more proficient in perspective and role taking, emotional expression, and social awareness. They develop greater self-awareness and the ability to manage their emotional expression and social behavior [16]. Based on the existing literature, it can be proposed that empathy-related constructs (such as empathy, personal distress, and fantasy) will be related to prosocial behavior in later adolescence.

Finally, one might ask how sociodemographic factors (such as age, gender, and level of parental education) relate to adolescent prosocial behavior and the attributes which may be associated with it (such as prosocial norms, pragmatic values, moral reasoning, and empathy constructs). In terms of age, since adolescents develop better moral reasoning as they get older, it may be expected that age is linearly related to prosociality. On the other hand, some theorists may also argue that an increase in age may not necessarily lead to an advancement of moral values, and there may be no relationship between these factors. Since there is not much research in this area, the present study attempts to examine the relationship between age and prosocial behavior. Furthermore, as females tend to show more empathy and to be more relationship oriented, it may also be expected that they will demonstrate higher levels of prosocial norms, moral reasoning and prosocial behavior, and lower levels of pragmatic values. Finally, as parental education implies greater social capital (such as better parenting and more involvement), it can also be predicted that parental education levels will be positively related to adolescent prosocial behavior. Against this background, the specific objectives of this study were (a) to examine the correlations between basic demographic factors (age and gender) and adolescent prosocial behavior and its related attributes (including prosocial norms, pragmatic values, moral reasoning, and empathy-related constructs); (b) to study the correlates of prosocial behavior, including prosocial norms, pragmatic values, prosocial reasoning, and empathy-related constructs; (c) to identify the key predictors of prosocial behavior among late adolescents. A cross-sectional survey was conducted using a sample of high school students in Hong Kong.

## 2. Method

### 2.1. Participants

The participants in this study were recruited from a group of high school students (secondary four to six) who attended a one-day “Teen Talk” event organized by the Hong Kong Law Society. The event was titled “Love Yourself, Love Others,” and its purpose was to engage young people to discuss their core values as well as those of society in a one full day seminar. As well as this, the event attempted to increase participants' understanding of legal and social issues.

 A total of 533 participants completed and returned survey questionnaires through their schools, giving a response rate of around 35.5%. Fifteen questionnaires were not included in the analysis either because more than 10% of items were incomplete or because they were extreme outliers. After discarding these questionnaires, 518 remained in the dataset. The participants were full-time students aged 14–22 (M = 16.2, SD = 1.1). There were more females (69.6%) than males (30.4%). They were recruited from 36 secondary schools and studying in secondary 4 (54.0%), 5 (40.2%), or 6 (5.8%). When asked about their educational achievements compared to classmates, more than one-third (37.1%) regarded themselves as better than average, and around half (48%) said they were average. A large proportion of participants regarded their conduct as better than average (48%) or very much better than average (14.3%).

Most of the participants were the only child in their family (72.4%), and 23.7% had one sibling. The median education level of both respondents' parents was secondary three. Financially, only 7.8% (*n* = 46) of these households were supported by social security benefits. Most of the respondents' fathers were employed full time (85.6%) or part time (4.1%), with 50.8% of mothers working full time.

### 2.2. Procedures

Ethical approval for this study was obtained beforehand from the Departmental Research Committee of The Hong Kong Polytechnic University. After access had been obtained, the schools which had participated in the Teen Talk event helped to distribute the questionnaires, invitation letters, and consent forms. If potential participants were under 18, an invitation letter was also sent to their parents. Parents were asked to sign a consent form and return it to the school. All participants were also requested to sign a consent form for voluntary participation. The schools sent the completed questionnaires and consent forms back to the researchers.

### 2.3. Instruments

#### 2.3.1. Prosocial Behavior

The adolescent behavior questionnaire (ABQ) is a generic Chinese-language instrument designed to measure the pro- and antisocial behavior of adolescents [17]. Respondents are asked to report the frequency of 65 behaviors performed in the past year on a 7-point Likert-type scale (1 = none, 2 = 1-2 times, 3 = 3-4 times, 4 = 5-6 times, 5 = 7-8 times, 6 = 9-10 times, and 7 = more than 10 times). The ABQ has two general subscales: the antisocial/delinquent behavior (DB) and prosocial behavior (PB) scales. The PB scale assesses normative and altruistic acts, while the DB scale covers rule-breaking, challenging, or aggressive behavior at school, home, and in social settings. The ABQ total score is the difference between the mean scores of the PB and DB scales. It indicates how far a young person's behavior is anti- (negative scores) or prosocial (positive scores).

#### 2.3.2. Prosocial Norms

Three items were taken from the Chinese youth positive development scale (CYPDS) to measure how willing participants were to provide help to the needy, participate in volunteer work and follow school rules. The reliability and validity of the CYPDS have been demonstrated in previous validation studies [18, 19].

#### 2.3.3. Pragmatic Values

The items used in a youth opinion poll titled “Young People's Outlook on Life” [20] were used here to assess respondents' pragmatic values. Respondents were asked to rate how far they agreed with eight pragmatic values in the local culture (such as “money can purchase happiness” and “a person must work very hard in order to be successful”). In the present study, factor analysis revealed two stable factors which explained 47.51% of the total variance. The first had two items (factor loadings ranging from  .62 to  .78) which focused on intention to abide by the law. The second comprised seven items (factor loadings ranging from  .53 to  .69) which focused on values reflecting a materialistic and “smart” mentality—in other words, pragmatic values. The second factor (containing seven items) is used in this study to indicate how far the respondent identifies with pragmatic values.

#### 2.3.4. Prosocial Reasoning

The Chinese version of the prosocial reasoning objective Measure (PROM) was used to assess participants' prosocial reasoning. The PROM is a measure assessing prosocial moral reasoning in young people and adults [21]. Respondents are invited to read stories about people who need help from others and then decide whether they would offer such help and the reasoning behind their response. The dilemmas in the stories are designed to invoke a conflict between the actor's needs, wants, and desires and those of another (or others). The Chinese short version uses five stories translated from the short version of the English PROM. After reading each one, the respondents are asked to rate on a scale from 1 to 5 (i.e., from most to least important) how important each of the five reasons is in deciding what the character should do. The five reasons reflect the five types of prosocial reasoning: hedonistic, needs oriented, stereotyped, approval oriented, and internalized. An overall weighted PROM score provides an indicator of the development of the respondent's prosocial reasoning. The PROM stories can be slightly modified for use with different age and cultural groups [22]. Its psychometric properties have been reported in studies based on participants from middle childhood to early adulthood with promising results [13]. The test-retest reliability of PROM ranged from  .70 to  .79, while Cronbach's *α* ranged from  .56 to  .78 [21].

#### 2.3.5. Empathy

The 21-item Chinese interpersonal reactivity index (C-IRI) is a self-report questionnaire consisting of three subscales: fantasy (FS), empathy (ES) and personal distress (PD). The participants were asked to indicate the degree to which each item described them using a 5-point Likert-type scale, which varied from 0 (does not describe me well) to 4 (describes me very well). A higher score in a subscale represents a higher functioning in each aspect of empathy. The C-IRI has acceptable psychometric properties in Chinese adolescent samples [23] and can be used as a valid instrument for assessing empathy.

## 3. Results

### 3.1. Sociodemographic Correlates of Prosocial Behavior

A multivariate analysis of variance (MANOVA) was conducted to examine gender and age differences in prosocial behavior and its related attributes. The sample was stratified into 4 age groups: 14-15 (*n* = 157), 16 (*n* = 191), 17 (*n* = 104), and 18 and above (*n* = 62) (4 cases had missing age data). The results showed that there was a significant gender difference (Wilks' Lambda = 9.59, *P* < .001) but no significant differences between age groups (Wilks' Lambda = 1.58, n.s.).

Further analyses using univariate ANOVAs showed that males reported more antisocial behavior than females, but there were no significant gender differences in prosocial behavior. However, using the overall ABQ scores as the outcome indicator, females were significantly more prosocial than males (*F* = 23.77, *P* < .001). Consistent with our predictions, females had higher levels of prosocial norms (*F* = 12.79, *P* < .001), pragmatic values (*F* = 6.67, *P* < .05), and prosocial reasoning (*F* = 20.47, *P* < .001). Furthermore, they had higher levels of empathy than males in several empathy-related constructs, including personal distress (*F* = 20.03, *P* < .001), fantasy (*F* = 11.01, *P* < .01), and empathetic concern (*F* = 10.92, *P* < .01). These findings are shown in Table 1.

### 3.2. Correlations of Prosocial Behavior

Correlation analyses were conducted to identify potential predictors of prosocial behavior (Table 2). Both the PB and overall ABQ scores showed a similar pattern of correlations, with the latter having a generally higher correlation with the predictors than the former. As well as this, prosocial norms, pragmatic values, prosocial reasoning and different constructs related to empathy were all associated with prosocial behavior and/or overall ABQ scores. As a result of this analysis, prosocial norms, pragmatic values, overall weighted PROM total, and the ES and PD subscale scores from the C-IRI were selected as potential predictors in the regression analysis. Parental education, calculated as the mean level of education of fathers and mothers, was not included in the further analysis because of the large amount of missing data (*n* = 454, *n*_missing_ = 64). Many participants appeared to be unsure about their parents' educational background.

### 3.3. Predictors of Prosocial Behavior in Adolescents

The findings on the predictors of prosocial behavior in adolescents are shown in Table 3. The predictors were able to predict a significant proportion of the variance in prosocial behavior as represented by the ABQ score (*R* ^2^ = .31, adjusted *R* ^2^ = .30). All variables, except the PROM score (*P* = .24), were found to be significant predictors of ABQ score. Comparing the sizes of *β*'s, the relative importance of the predictors is as follows: prosocial norms (*β* = .32), pragmatic values (*β* = −.19), empathetic concern (*β* = .18), and personal distress (*β* = −.14). Increased scores in prosocial norms and empathy, and decrease in pragmatic values and personal distress, are associated with increases in prosocial behavior. Preliminary collinearity analysis using tolerance and VIF revealed no major concerns. However, analysis using the condition index showed that the PROM score shared significant variance proportions with both empathy and prosocial norms in two dimensions. Multicollinearity among these three variables may therefore force the PROM score to become an insignificant predictor. Nevertheless, removing it did not result in a significant reduction in the accuracy of the prediction (*R* ^2^ = .30, adjusted *R* ^2^ = .29).

There were some differences in the results of the regression analyses conducted for the male (*n* = 155) and female (*n* = 355) subsamples (see Table 4). Prediction was stronger for girls (*R* ^2^ = .34, adjusted *R* ^2^ = .33) than boys (*R* ^2^ = .29, adjusted *R* ^2^ = .27). The significant predictors for females were the same as those for the whole sample, but only personal distress, empathetic concern, and prosocial norms were significant for the male subsample.

## 4. Discussion

In terms of sociodemographic correlates, while the findings do not reveal any age effect, they demonstrate significant gender differences in ABQ scores, prosocial norms, pragmatic values, prosocial reasoning, and empathy (personal distress, fantasy, and empathetic concern). These findings are generally consistent with those reported in the literature as well as with the hypotheses of this study.

Consistent with the original expectations, several factors are related to prosocial behavior in both the correlation and regression analyses. Higher levels of empathy and prosocial norms as well as a lower level of pragmatic values are associated with a higher level of prosocial behavior. These three predictors remain significant in separate regression analyses with the male and female subsamples. These results are highly consistent with theoretical predictions and previous empirical results [24, 25]. They also underscore the role of pragmatic values as a predictor of prosocial behavior, which has seldom been explored in previous studies. The present study suggests agreement with pragmatic values (associated with materialism and self-centeredness) could hamper prosocial behavior. Based on this conjecture, further work should be conducted to examine how different values may promote or inhibit prosociality.

The present findings indicate a significant association between personal distress and prosocial behavior, but the relationship is negative rather than positive in nature. Most previous studies show that higher levels of personal distress and sympathy are associated with more prosociality towards persons in need [26, 27]. The result in this study contradicts the proposed hypothesis as well as previous research findings. Since empathy is a multidimensional construct, while sympathy, personal distress, and perspective taking may make unique contributions to prosocial behavior, they can also interact with each other in predicting it [28]. For example, while personal distress may elicit a motivation to help, it could also inhibit prosocial actions when fear, tension, or distress is too high. As such, further research is needed to examine the relationship between empathy-related responses and prosocial behavior.

While prosocial reasoning as assessed by the PROM score is correlated significantly with prosocial behavior, it fails to predict prosocial behavior in the regression analyses. This may be due to the significant collinearity among prosocial reasoning, empathy, and prosocial norms. Analysis using condition indices shows that these three variables share significant variance proportions in several dimensions. Nevertheless, it is noteworthy that prosocial reasoning only explained a small proportion of variation in prosocial behavior (4.5%) when it was entered as the only predictor in the regression.

The four significant predictors, including empathetic concern, prosocial norms, pragmatics values, and personal distress, were able to predict around 30% of variation in prosocial behavior. The variance explained was a bit higher for the female (33%) than male (27%) sample. The percentage of variance explained was statistically significant, but much of the variation was not accounted for. In future studies, it will be a good idea to add predictors involving social influences such as peer and teacher influence, parent and school socialization, school discipline and encouragement for prosocial involvement, or interpersonal competence [9, 29].

These findings should be interpreted with reference to several factors. Firstly, while the sample was large, it was a convenience sample recruited from high schools with the age range largely limited to late adolescence (age 15–18). Secondly, the students involved had been nominated by their schools to take part in the “Teen Talk.” Since it is likely that schools will prefer to have students with better academic or conduct records to take part in these types of community events, the sampled group may have displayed better previous conduct. Thirdly, we found that the range of prosocial behaviors in the ABQ could be expanded further. While it covers prosocial acts in home, school and social situations, additional items could be added to sample a wider range of prosocial behaviors like providing support or assistance to people one knows or does not know: sharing, listening, and comforting; appreciating others; working in a team; involvement in prosocial groups (such as service teams or religious groups) [30].

In summary, this study has shown that empathetic concern, personal distress, prosocial norms, and pragmatic values are key predictors of prosocial behavior among older adolescents in Hong Kong. These results are largely consistent with theoretical expectations and the findings of previous work, other than the negative relationship between personal distress and prosocial behavior. Further study is needed to examine the unique and combined effect of empathy-related constructs (empathy, sympathy, perspective taking, and personal distress) on prosocial development. This study also provides support for the importance of values and norms in predicting prosocial behavior, which has seldom been explored in previous studies. While prosocial reasoning shows a significant correlation with prosocial behavior, it is not a significant predictor of it. This is probably a result of the collinearity of prosocial reasoning with empathy and prosocial norms. In further studies looking at predictors of prosocial behavior, it will be necessary to design or employ a standardized measure that provides a wider coverage of such activity. It will also be necessary to select and measure additional predictors which reflect peer, family, and school influences on prosocial development.

## Figures and Tables

**Table 1:** Sex differences in prosocial behavior and its correlates.

Variable^a^	Sex	M	SE	95% CI		*F*	*η* ^2^
				Lower	Upper		
Antisocial behavior	M	3.46	.22	3.03	3.88	20.64***	.04
	F	2.25	.15	1.95	2.55		

Prosocial behavior	M	3.31	.14	3.02	3.59	.49	.00
	F	3.43	.10	3.23	3.63		

ABQ score	M	−.15	.22	−.59	.29	23.77***	.03
	F	1.18	.16	.87	1.49		

Prosocial norms	M	4.65	.05	4.54	4.75	12.79***	.03
	F	4.88	.04	4.81	4.95		

Pragmatic values	M	2.72	.04	2.63	2.81	6.67*	.01
	F	2.86	.03	2.80	2.92		

PROM overall weighted	M	6.46	.08	6.31	6.61	20.47***	.04
	F	6.88	.05	6.77	6.99		

Personal distress subscale	M	1.98	.05	1.88	2.07	20.03***	.06
	F	2.28	.03	2.22	2.35		

Fantasy subscale	M	2.07	.06	1.95	2.19	11.01**	.02
	F	2.32	.04	2.24	2.41		

Empathy subscale	M	2.45	.04	2.36	2.52	10.92**	.02
	F	2.60	.03	2.54	2.65		

Note: ^a^estimated marginal means are shown. *P < .05; **P < .01; ***P < .0001.

**Table 2:** Correlations between potential predictors and adolescent antisocial and prosocial behavior.

Potential predictors	Antisocial behavior	Prosocial behavior	Adolescent behavior
Age	.06	.05	−.02
Parent education	−.08	.18***	.19***
Prosocial norms	−.28***	.30***	.46***
Pragmatic values	.32***	−.09*	−.36***
PROM overall weighted	−.16***	.09*	.21***
Personal distress subscale	.11*	.02	−.09*
Fantasy subscale	.10*	.14***	−.00
Empathy subscale	−.18***	.32***	.39***

**Table 3:** Prediction of prosocial behavior (ABQ) among adolescents (*N* = 516).

Predictors	*b*	SE	*β*	*t*	*P*	Collinearity statistics	
						Tolerance	VIF
Pragmatic values	−.90	.20	−.19	−4.52	<.001	.81	1.23
Prosocial norms	1.28	.17	.32	7.53	<.001	.75	1.33
Prosocial reasoning (PROM overall weighted)	.13	.11	.05	1.17	.24	.84	1.19
Empathy subscale	.98	.25	.18	3.97	<.001	.68	1.46
Personal distress subscale	−.63	.17	−.14	−3.59	<.001	.92	1.08

Note: *R* ^2^ = .31, adjusted *R* ^2^ = .30.

**Table 4:** Prediction of prosocial behavior (ABQ score) among males and females.

Variables		*b*	SE	*β*	*t*	*P*
Males	Personal distress	−1.45	.35	−.29	−4.12	<.001
	Empathy	1.05	.46	.17	2.27	.02
	Prosocial norms	1.15	.27	.31	4.30	<.001
	PROM overall weighted	.19	.19	.07	1.00	.32
	Pragmatic values	−.60	.34	−.13	−1.77	.08

Females	Personal distress	−.48	.20	−.11	−2.37	.02
	Empathy	.82	.29	.16	2.81	.01
	Prosocial norms	1.48	.22	.35	6.77	<.001
	PROM overall weighted	.04	.14	.01	.26	.80
	Pragmatic values	−1.02	.24	−.21	−4.25	<.001

Note: For male subsample (*n* = 155), *R* ^2^ = .29, adjusted *R* ^2^ = .27; for female subsample (*n* = 355), *R* ^2^ = .34, adjusted *R* ^2^ = .33.
